# Nutraceuticals, Social Interaction, and Psychophysiological Influence on Pet Health and Well-Being: Focus on Dogs and Cats

**DOI:** 10.3390/vetsci12100964

**Published:** 2025-10-09

**Authors:** Mario Nicotra, Tommaso Iannitti, Alessandro Di Cerbo

**Affiliations:** 1 School of Biosciences and Veterinary Medicine, University of Camerino, 62024 Matelica, Italy; mario.nicotra@unicam.it; 2 Section of Experimental Medicine, Department of Medical Sciences, University of Ferrara, Via Fossato di Mortara 70, 44121 Ferrara, Italy; tommaso.iannitti@gmail.com

**Keywords:** pets, longevity, nutraceuticals, social interaction

## Abstract

**Simple Summary:**

Pet humanization has transformed animal healthcare and highlighted the importance of nutrition in promoting human–pet social interaction, animal psychophysical well-being and, possibly, longevity. This review examines the impact of nutraceuticals, such as omega-3 fatty acids, pre- and probiotics, plant extracts and dietary supplements in enhancing the human–animal bond by reducing stress and modulating the gut–brain axis, managing bone, skin, and immune diseases and even gastrointestinal disturbs.

**Abstract:**

Pet humanization, particularly in dogs and cats, has transformed animal healthcare and highlighted the importance of nutrition in promoting human–pet social interaction, pet psychophysical well-being and, possibly, longevity. Nutraceuticals, such as omega-3 fatty acids, prebiotics, probiotics, plant extracts and dietary supplements, are endowed with antioxidant, anti-inflammatory, immune-modulating, cognitive-enhancing and gut-microbiota balancing properties. These effects have been shown to contribute to the possible prevention and management of bone and skin diseases, as well as gastrointestinal and behavioral disturbs. Moreover, the human–animal bond has been shown to play a pivotal role in reducing stress, improving sociability, and modulating pets’ emotional and physiological states. Evidence also suggests that nutrition and social interactions can influence the gut–brain axis, impacting the behavior, cognition, and resilience to stress-related disorders. Besides underlining the value of nutraceutical integration into pet nutrition strategies and offering a comprehensive, evidence-based perspective on their potential in improving animal welfare, literature reports about drawbacks of the use/misuse of such substances have been reported.

## 1. Introduction

The global pet population is estimated to be approximately 703.3 million individuals [1,2]. A recent key factor contributing to the rise in the pet population was the COVID-19 global pandemic, as interest in pet adoption, primarily of dogs and cats, increased during lockdowns [3].

This growth reflects a substantial shift in the human–animal bond. Indeed, whereas domestic animals were once mainly seen as mere working animals, such as watchdogs or mousers, they are now perceived as integral family members and therefore humanized [4,5,6]. Consequently, pet owners are increasingly concerned about their pets’ health and dietary habits [7], paying more attention to the ingredient list and manufacturing process [8], continuously seeking out the best food products that combine high quality, safety, and adequate nutritional value [9,10].

The “anthropomorphization” of pets [11] also contributed to the spread of alternative dietary regimes, including homemade as well as plant- and raw meat-based diets [12], probably due to two main factors: a growing wariness against commercial pet food and the pet food industry, and the enjoyment from preparing food for their pets [13]. In addition, a growing interest in natural ingredients has also been registered, as the inclusion of natural products is widely regarded as a reliable indicator of quality [14].

In this sense, nutraceuticals have gained increasing attention in human [15] nutrition for their safety, nutritional profile, and potential therapeutic applications. This neologism refers to a group of compounds that confer health benefits and play a role in the prevention, management, or treatment of illness [16,17,18,19].

Nutraceuticals are from natural origin (mostly plants, algae, or fungi) and include vitamins [20,21,22], minerals [23], healthy fatty acids [24], polyphenols [25,26,27], glucosinolates [28], terpenoids [29], and alkaloids [16,30]. They have become popular in human nutrition due to their positive influence in several pathological contexts such as diabetes [31], cancer [32,33,34], atherosclerosis [35,36], as well as cardiovascular [17,37] and neurological [18,38] disorders.

Similarly, over the past three decades, their popularity has also increased in veterinary medicine [39], particularly in dogs, cats, and horses [40,41], with successful results on skin [42,43] and ocular disorders [44,45], auricular issues [46], behavioral [47,48] and reproductive [49,50] disturbances, as well as chronic pathologies [51,52,53,54,55]. The great potential and importance of nutraceuticals have also been perceived by pet food industry, which has started incorporating them into pet food formulations. This has created a market with an estimated value of nearly $6 billion in 2023, projected to exceed $10.5 billion by 2035 [56,57]. Concurrently, veterinarians have progressively understood their worth, too, and often employ them, alone or in conjunction with pharmaceutical drugs, to guarantee their clients’ and their own animals’ well-being [58].

This review aims to highlight the main nutraceutical compounds in veterinary medicine and their benefits for companion animals’ health as well as their potential in possibly improving the social interactions.

### 1.1. Diet and Social Interactions

The social perception of animals has changed over time. In contemporary society, companion animals, particularly dogs and cats, are recognized as integral members of the family [5,6]. Their owners establish a profound emotional bond with them, whose intensity is comparable to that shared with human family members [59].

This connection exhibits a dualistic nature, holding both positive and negative aspects for humans and animals. On the one hand, pets receive food, water, and protection from pain, and diseases [5], while owners, in turn, receive love, physical and psychological benefits [59,60,61]. This aspect led to the creation of the term “Zooeyia” [62], which refers to all the health benefits humans gain from interacting with their companion animals. On the other hand, sometimes the owners run the risk of overhumanizing their pets, which can lead to serious issues, such as wrong feeding habits and onset of obesity and diabetes, and welfare impairment, dressing pets or failing to give them enough physical activity [61].

In this sense, food can play a crucial role in the pet-owner relationship, not only as a means to show love and affection but also as a source to compensate potential nutritional deficits [63,64,65]. Pets supplemented with a high-quality diet can positively improve their sociability, making them more prone to human contact and reducing behavioral issues, especially in dogs [66]. Food can also strengthen the bond between pets and their owners [67] or serve as a positive reinforcement to reward desirable behaviors [68].

Furthermore, it can be strategically employed to mitigate behaviors that disrupt cohabitation between owners and their pets, such as cats’ nocturnal activity [68], thereby improving the overall quality of shared living. In this context, nutraceuticals hold great potential in managing specific pet conditions, e.g., halitosis, that pose a significant problem in the social interaction with their owners [53]. For instance, the supplementation with a nutraceutical diet including *Ribes nigrum*, *Salvia officinalis*, *Thymus vulgaris*, and egg albumen effectively relieved halitosis in dogs [53,54]. Another factor that could seriously impact pet-owner relationship is stress [69,70], a complex response of the organism following exposure to a hostile environment or noxious physical or psychological stimuli [71,72]. It is commonly associated with behavioral expressions, including auto-grooming, body shaking, defecating, urinating, circling, floor licking, and vocalizing, that may alter the daily social equilibrium in the long run [73,74].

In this regards, chewable tablets with *Rhodiola rosea* and *Passiflora incarnata*, niacinamide, phospholipids, L-tryptophan, and thiamine hydrochloride, significantly improved stress-related responses in dogs with a behavioral history of stress by significantly reducing the mean stress severity score per stressor and noise sensitivity [70]. It can be therefore argued that food can contribute to the strengthening the pet–owner relationship but also help in overcoming some annoying behaviors that may threaten the solidity and durability of this highly complex bond.

### 1.2. Diet and Longevity

Improving health and while, possibly, extending longevity have become of utmost importance for scientists in both humans and pets [75,76,77]. Adopting a healthy lifestyle, including balanced dietary habits, is crucial to prevent the onset of chronic diseases and extend lifespan, ensuring a “healthy longevity” [78,79,80].

In recent decades, there has been a notable increase in the life expectancy of companion animals, thanks to advancements in veterinary medicine and nutrition, as well as the adoption of more responsible ownership practices [81]. Adequate nutrition, along with supplementation of nutraceuticals, have been associated with reduced onset of diseases as well as improved sociability and quality of life, thus possibly contributing to longevity of pets [76,77,82,83,84].

Dietary interventions in cats and dogs have been shown to delay the aging process and prevent specific age-related metabolic alterations, particularly those affecting the renal, pulmonary, cardiovascular, and gastrointestinal systems, thereby improving their health and extending their lifespan [81,85,86,87,88].

Among the various dietary strategies employed to enhance lifespan, caloric restriction (CR), for instance, has been linked to improved health outcomes and increased longevity [89,90] through multiple mechanisms, including a reduction in oxidative damage [91], a decrease in body temperature [92], and modulation of sirtuin activity [93]. In this regard, polyphenols, alkaloids, carotenoids, and hormones have been shown to modulate the same cellular and physiological pathways involved in CR, thereby mimicking its beneficial effects and potentially promoting longevity [94].

Also, oils, extracts, or other derivatives from different plants, such as flaxseed, sunflowers, Black Ginseng, *Yucca schidigera*, chestnut, curcumin, and fructans, significantly improved the well-being, and the health of gastrointestinal, immune, and cardiovascular systems [94,95,96,97,98,99,100,101,102,103].

Other nutraceuticals, such as resveratrol, hawthorn leaves flavonoids, grape seed proanthocyanidin, tea polyphenols, and *Rhus verniciflua*, resulted effective in counteracting pathological conditions such as poisonings, metabolic, vascular, and digestive issues [104,105,106,107,108]. Consequently, all these favorable outcomes positively improved pet’s behavior and, in turn, their relationship with owners.

### 1.3. Human–Animal Bonding and Pets’ Well-Being

The first studies concerning the impact that interacting with humans has on animals date back to the late ‘60s, and were conducted on experimental animals [109,110]. In 1966, Lynch and McCarthy exposed nine dogs to human presence and a stressor stimulus (i.e., electrical stimulation) and observed a decrease in heart frequency and the suppression of the foot flexion reflex if the animal was petted for 10 s while receiving the stressor [110]. Both signs were associated with a positive impact on human-pet interaction, possibly indicating an animal more prone to establish a relationship. Being more than an isolated case, the study from Lynch and McCarthy fostered similar future studies, which markedly raised the interest in societal perception of pets in recent decades, thus emphasizing the respective positive impact of human–animal interaction. Simple acts, such as petting, training, or playing with them, can help pets alleviate their stress [111], possibly modulating also some behavioral disorders including anxiety, fear, and hyperactivity [48]. The stress-attenuating effect due to the interaction was also evident while assessing the impact of verbal and tactile interaction between owners and their pets during a veterinary clinical examination [112]. Results revealed a reduction in examination-related stress, as indicated by fewer attempts to jump off the exam table, a lower heart rate, and a lower maximum ocular surface temperature in dogs receiving tactile and verbal support from their owners.

A similar positive impact on stress was observed in sheltered animals [113,114]. Two in vivo studies found that human interaction could reduce stress in these animals, as confirmed by improvements in sociability, diffidence, temperament, excitation, and vocalization, and panting, followed by reductions in cortisol levels in blood plasma and saliva [113,114].

Human-pet bond can also influence the blood concentration of oxytocin, a pivotal hormone involved in the establishment and duration of the relationship [115], whose increased and rapid release is a consequence of positive emotions such as affection (i.e., kisses), love and physical interaction [116,117,118].

Similarly to dogs, the interaction with humans has also been linked to reduced stress and anxiety in cats. It has been proposed as an alternative strategy to mitigate stressful behaviors, and research has shown that gentling can also enhance the production of S-IgA and prevent the onset of upper respiratory diseases [119,120,121]. All this evidence supports the importance that the human–animal bond holds not only for human health but also for the well-being of companion animals. Thus, emotional factors such as love and care can positively affect the well-being of humans and pets by decreasing stress and, if combined with a diet enriched with antioxidants, improving the quality of life and promoting longevity.

## 2. Materials and Methods

This review was conducted to comprehensively and critically summarize the scientific literature, examining the role of nutraceuticals in enhancing pet longevity and influencing social behaviors and interactions with humans. Relevant peer-reviewed publications were identified through searches of electronic databases, including PubMed, Web of Science, Scopus, and Google Scholar, covering the period from January 2000 through June 2025. Search terms combined key concepts related to companion animals, nutraceuticals, longevity, aging, cognition, and social behavior, including: “nutraceuticals” AND “pet food”, “functional ingredients” AND “dogs AND cats”; “dietary supplements” AND “companion animals” AND “Animal welfare” AND “Emotional bonding” AND “Proper animal care” AND “Social interaction” AND “Physical activity support” AND “Quality of life improvement” AND “Antioxidant protection” AND “Cellular protection” AND “Longevity promotion” AND “Omega-3 fatty acids” AND “Anti-inflammatory support” AND “Balanced phosphorus” AND “Renal (kidney) health” AND “Holistic nutrition” AND “Integrated care” AND “Nutritional balance” AND “Synergistic effects of nutrients” AND “longevity”. Boolean operators (“AND,” “OR”) were used to refine the queries. Additional references were retrieved by cross-referencing citations in relevant articles and reviews.

Based on the results retrieved from the literature, we first summarized the mechanism of action, scientific evidence, species-specific considerations, and commercial availability of the main nutraceutical substances used in the pet food industry, such as omega-3 fatty acids, prebiotics and probiotics, antioxidants, plant-based extracts, and dietary supplements such as vitamins, minerals, glutathione, glucosamine, and chondroitin sulphate.

Then, we described their health benefits in the main pet apparatuses, such as joints, skin and coat, gut, cognitive functions, and immunomodulation related to pet longevity and social interactions.

## 3. Results and Discussion

### 3.1. Omega-3 Fatty Acids

Polyunsaturated fatty acids, or “PUFAs”, consist of a long chain of carbon atoms, a carboxyl group on one end, and a methyl group on the other. They exhibit at least two double bonds between the carbons of the chain and are classified as Omega-3 (n-3) and Omega-6 (n-6) fatty acids, respectively, based on the presence of a double bond on the third or sixth carbon atom from the methyl end [122]. N-3 fatty acids can be further distinguished into short-chain (SC), such as alpha-linolenic acid (ALA; 18 carbon atoms), or long-chain (LC), including eicosapentaenoic (EPA; 20 carbon atoms) and docosahexaenoic (DHA; 22 carbon atoms) acids [122,123].

ALA is abundant in plant oils, such as flaxseed, walnuts, soybeans, hemp, rapeseed, chia, canola, and perilla oils [124,125], while fish and seafood are rich in EPA and DHA [126]. In humans as well as in mammalian species, ALA can be converted into EPA and DHA [127]. However, this conversion is not efficient enough to meet the adequate requirements for EPA and DHA, which are considered essential nutrients and must be supplied through the diet [120,121,122,123,124,125,126,127,128,129,130,131].

#### Health Benefits of Omega-3 Fatty Acids and Mechanisms of Action

Omega-3 fatty acids offer several health benefits for humans and animals, which can help increase their lifespan and promote longevity, while also enhancing their overall quality of life. First, they are endowed with anti-inflammatory and immune-modulating properties [132]. These activities can be related to several mechanisms, including the modulation of inflammatory mediator production, such as eicosanoids, tumor necrosis factor alpha (TNF-α), ad interleukin 6 (IL-6) [133], and the impairment of leukocyte adhesion to endothelium due to the reduced expression of adhesive surface molecules on endothelial cells [134,135], monocytes [136], macrophages, [137] and lymphocytes [138]. In addition, another hypothesized anti-inflammatory mechanism is related to the production of n-3 PUFAs metabolites (resolvins D and E, marensins, and protenectins) by specific enzymes such as lipoxygenases and cytochrome P450 [139].

The anti-inflammatory effects of n-3 PUFAs may also be responsible for the amelioration of clinical conditions and the faster reduction in carprofen need in dogs with osteoarthritis [140,141].

Omega-3 fatty acids also support the health of skin and hair coat, especially in animals suffering from allergies or other inflammatory skin conditions, by competing with arachidonic acid (AA) for cyclo-oxygenase and 5-lypoxygenase and leading to the production of eicosanoids that exert anti-inflammatory activity or have weaker pro-inflammatory activity than the ones derived from AA [142,143,144]. Moreover, high DHA was observed to reduce excessive skin oil secretion, thereby supporting coat quality [145].

They can also enhance cardiovascular functions through various mechanisms. For instance, they have antiarrhythmic properties that are likely due to their modulation of ionic currents, including L-type calcium currents, in cardiac cells [146,147]. They can also reduce blood pressure and protect against ischemic damage by modulating leukocyte function and reducing their infiltration into ischemic tissue [146,148].

In addition, n-3 fatty acids impact reproduction by influencing the production of steroid hormones, modulating the secretion of molecules involved in reproduction, such as eicosanoids like PGE_2_ and PGFα, and affecting the functionality of sperm and oocytes [149]. In females, they influence the development of oocytes and support the germinal vesicles, thereby preventing them from breaking down [150]. Moreover, they inhibit cyclooxygenase, modulating the production of prostaglandins, including PGE_2_ and PGFα, which play a key role in the development, maintenance, and regression of the corpus luteum. In males, on the other hand, n-3 fatty acids have been associated with the improvement of several characteristics of spermatozoa, including increased membrane fluidity, motility, morphology, and concentration [151]. On the contrary, dietary restriction of n-3 PUFAs was linked to poor semen quality, and reduced levels of EPA and DHA were found in sperm samples from infertile patients [49,151].

Omega-3 supplementation has also been shown to exert renoprotective effects in dogs by counteracting hypercholesterolemia and hypertriglyceridemia, both of which had progressively negatively impacted renal health [152]. Moreover, these protective effects can also be attributed to the omega-3-induced increase in glomerular filtration rate (GFR), reduction in oxidative damage, and suppression of inflammation [153].

Finally, n-3 PUFAs also exert several benefits on brain development and vision, both in young and aging patients [154]. DHA concentrations increase during perinatal brain development, reaching around 1020% of its total fatty acid composition 21 days after birth. They support the maturation of the brain, enhancing the formation of synapses and the development of neuronal arborization [155]. Furthermore, EPA and DHA have been shown to positively affect cognition in aging dogs and cats, likely due to their protective effects against inflammation and oxidative stress, as well as their ability to enhance neurogenesis and glucose transporter activity [156]. However, omega-3 supplementation is not free from side effects. Indeed, research reports potential adverse effects, including weight gain, impact on insulin sensitivity, alterations in platelet function and wound healing, diarrhea, pancreatitis, and lipid peroxidation, even though studies are conflicting. Moreover, Omega-3 supplementation can increase the risk of falling into fat-soluble vitamins hypervitaminosis, alterations in immune function, with decreased neutrophil function and lymphocyte counts, and a negative impact on hypersensitivity reactions and nutrient–drug interactions, especially when administered concurrently with NSAIDs, such as Carprofen [29].

All this evidence shows the Importance of omega-3 PUFAs in companion animals’ health and their potential to prevent the onset of pathological conditions.

### 3.2. Prebiotics and Probiotics

The term “probiotics” refers to live microorganisms that positively affect the host’s health if supplemented in proper amounts [157,158]. They primarily act by improving intestinal microbial balance, leading to both intestinal and gastrointestinal benefits, including immune-modulating [157]. The most popular probiotics for dogs and cats come from the *Lactobacillus* and *Bifidobacterium* genera (e.g., *Bifidobacterium animalis* subspecies *lactis* CECT 8145 [159], *Bacillus velezensis* DSM 15544 [160]). Recently, however, researchers have begun investigating yeasts and other microorganisms, such as *Saccharomyces cerevisiae* and *Akkermansia muciniphila*, *Faecalibacterium prausnitzii*, *Eubacterium hallii*, *Prevotella copri*, *Christensella minuta*, *Parabacteroides goldsteniii,* and species belonging to the *Bacteroides* genus for their probiotic properties [161,162,163,164]. Many of the prebiotics used to ensure the health and well-being of pets are of human origin [165,166]. Nevertheless, the focus of research has shifted to isolating potential probiotic strains from animal hosts, aiming to enhance further their safety and efficacy in promoting health in companion animals [167,168].

Prebiotics, on the other hand, are non-digestible nutrient substrates selectively employed by the microorganisms present in the host’s gut microbiota. Their degradation products, mainly short-chain fatty acids, enter the bloodstream, thereby potentially extending their health benefits to other districts beyond the gastrointestinal tract [168,169,170].

The most common prebiotics employed in animal nutrition include fructans, such as fructooligosaccharides (FOS) and inulin, galactans, including galactooligosaccharides (GOS), Xylooligosaccharides (XOS), lacticol, and cereal fibres [171].

Pre- and probiotics work synergistically; as a result, they are frequently combined in formulations known as “synbiotics” [172,173].

#### Health Benefits of Pre- and Probiotics and Mechanisms of Action

Ensuring an optimal balance within the gut microbiota is crucial for maintaining the host’s health [174]. Alteration to its homeostasis, known as “dysbiosis”, can significantly affect an animal’s well-being and increase the risk of developing pathological conditions, including inflammatory bowel disease (IBD), obesity, or neurological disorders [175].

Companion animals can benefit positively from pre- and probiotic supplementation. Prebiotic supplementation contributes to intestinal health by promoting the proliferation and activity of beneficial intestinal microbes, such as bifidobacteria and lactobacilli, thus positively impacting gut microbiota. Moreover, they can also be employed to modulate fecal pH, consistency and bulk, and increase short-chain fatty acids (SCFA) production [176]. In dogs, as in humans, gut microbes can ferment prebiotics, thereby producing acetate and lactate that serve as substrates for other microbial species, which utilize them to produce butyrate, another SCFA.

Butyrate represents the primary energy source for colonocytes, contributes to intestinal well-being, and exhibits immunomodulatory properties by modulating the production of IL-2 and IL-6 [177].

Prebiotics can also play a role in the prevention of certain disorders, such as obesity, which could potentially facilitate the onset of chronic pathologies, including diabetes mellitus. Indeed fibers, particularly those with high fermentability, can enhance satiety, thereby reducing daily energy intake and promoting weight management [178]. Moreover, prebiotics can positively impact insulin sensitivity, glucagon-like peptide-1 (GLP-1) secretion, and postprandial hyperglycemia [179,180], thus representing a potential means to prevent the onset of diabetes. In addition, despite their capacity to modulate gut microflora and restore SCFA production, prebiotics are often used, in combination with probiotics, to counteract gastrointestinal diseases and their clinical signs, including diarrhea [181].

Likewise, probiotic supplementation affects gut microbiota and plays a pivotal role in rebalancing the gut microbiota of companion animals with gastrointestinal disorders [161]. Probiotics enhance the host’s health through different mechanisms. For instance, they can interact synergistically with the gut microbiota, supporting its stability and cooperating in the metabolization of nutrients. They can also prevent pathogens from colonizing the gut by stimulating the synthesis of antimicrobial compounds, such as bacteriocins, and by competing for nutrients and sites, thereby exerting an overall protective action [182]. Moreover, they can ameliorate the intestinal barrier integrity by stimulating mucin production, promoting the well-being of intestinal epithelial cells, and reducing the production of pro-inflammatory cytokines [183,184].

In both dogs and cats, probiotics have been shown to improve fecal quality and reduce fecal nitrogen fermentation products, ammonia emissions, fecal inflammatory markers, and overall fecal odor, while increasing fecal antioxidant and the production of SCFAs [185,186,187,188,189]. In addition, their potential therapeutic use has been proposed in the management of several gastrointestinal disorders, including colonic dysmotility [190] and diarrhea [191,192] in dogs. Furthermore, research in felines reports their positive impact on managing oral issues, such as stomatitis, gingivostomatitis, and both bacterial and viral oral infections, as well as improving immunity, hepatic, and renal health [193,194]. For instance, cats with chronic kidney disease (CKD) at stage 2 or 3 supplemented for two months with a *Lactobacillus* mixture showed reduced or preserved concentrations of the blood urea nitrogen (BUN) and creatinine, the two leading kidney function indicators in blood plasma, and indoxyl sulfate, a gut-related uremic toxin whose increase in circulation is negatively correlated with the progression of CKD. Furthermore, the treatment resulted in increased appetite, activity, and defecation frequencies, thereby contributing to an overall enhancement of the patients’ quality of life [194].

The influence of probiotics on microbiota composition can also indirectly impact animals’ behavior by affecting the bidirectional communication system, which links the intestinal and nervous systems, known as the “gut–brain axis” (GBA) [195].

Indeed, modifications in the intestinal microbial composition have been linked with behavioral issues, such as aggressiveness and anxiety [196,197,198]. In this context, probiotic supplementation has shown beneficial effects on modulating behavioral disorders, thus representing a promising nutraceutical option in the treatment of these conditions [199,200]. For instance, daily supplementation with *Lactobacillus plantarum* LP815^TM^ for four weeks improved aggressiveness and anxiety in 40 dogs effectively. There were no adverse effects, and sleep regularity was enhanced as an additional benefit [200].

The use of pre- and probiotics is generally considered safe. However, prebiotics misuse can lead to flatulence, diarrhea, and abdominal pain [201], while probiotic supplementation, although occasionally, can result in opportunistic infections, and in immunological and metabolic disturbances [202]. Furthermore, probiotics can carry antimicrobial resistance genes (ARGs), particularly those belonging to the genus *Enterococcus*, thus contributing to the spread of antimicrobial resistance (AMR) [202,203,204].

In any case, despite the absence of a specific recommendation, research findings indicate that the provision of pre- and probiotics to companion animals exerts a direct influence not only on their gastrointestinal health. Furthermore, it can indirectly engender extraintestinal favorable outcomes, such as those observed in behavior, promoting animal health and ensuring their longevity.

### 3.3. Plant Extracts

This increased interest in medicinal plants and herbal extracts stems from the perception that natural compounds are safer, more economical, and environmentally friendly than their synthetic counterparts, yet are just as effective [205]. Medicinal plants hold great potential in veterinary medicine because they produce phytocompounds, such as polyphenols (e.g., flavones, flavanones, and flavanols), carotenoids (e.g., lutein and lycopene), isoprenoids (e.g., limonene and pinene), phytosterols (e.g., campesterol and sitosterol), saponins (e.g., dammarane and oleanane), dietary fibers (e.g., pectin and cellulose), and specific polysaccharides (e.g., amylose and amylopectin) that confer several health-enhancing biological properties [206].

These phytochemicals are each associated with specific health-enhancing properties, including antimicrobial and immunomodulatory activities, stress relief, and growth promotion [207]. Polyphenols, for example, are secondary metabolites of plants and are abundant in foods of vegetable origin, such as fruit (e.g., blackberries, strawberries, kiwi, cherry, peach, apricots, apples, etc.), vegetables (e.g., aubergines, artichokes, potatoes, leeks, etc.), cereals, nuts, and legumes [208,209]. They are well known for their antioxidant activity, but they possess other bioactivities, including anti-inflammatory, cardioprotective, antineoplastic, and antimicrobial properties [210].

Carotenoids, such as β-carotene, are one of the primary dietary sources of vitamin A. They are abundant in fruits (e.g., apricots and peaches), green vegetables (e.g., spinach and broccoli), along with some foods of animal origin, including butterfat, egg yolk, and salmon. These compounds have been associated with several health-enhancing properties, including antioxidant, immunomodulatory, cardioprotective, and antineoplastic activities [211].

Phytosterols, on the other hand, are steroids of natural origin endowed with chemopreventive, antioxidant, anti-inflammatory, and antidiabetic effects [212], also able to impact the metabolism of cholesterol in humans and dogs [210,213].

#### Health Benefits of Plant Extracts and Mechanisms of Action

Plant extracts can be used either to improve the well-being of healthy animals, alone or in combination with other substances, by enhancing immune, gastrointestinal, and cardiovascular health, as well as contrasting pathological conditions such as heavy metal poisonings, metabolic issues, including obesity, and diseases involving the digestive tract [76]. The health benefits of several plant extracts have been investigated for their potential therapeutic use in veterinary medicine [214].

For example, Kim et al. (2020) assessed the anti-inflammatory potential in beagle dogs fed two different doses of black ginseng extract for 8 weeks by evaluating their serum metabolic profile [97]. Results have shown a statistically significant difference in the metabolic profiles of treated and untreated dogs, which is hypothesized to be linked to its biological activity. The anti-inflammatory effect is probably due to the presence of saponins, such as ginsenoside Rh_2_, Rg1, Rb1 and Rg3, that inhibit the production of some pro-inflammatory mediators, such as nitric oxide (NO), COX-2, and pro-inflammatory cytokines such as TNF-α and interleukin-1β (IL-1β), along with an increase in anti-inflammatory cytokines, specifically IL-10 [215,216].

Likewise, Reichling et al. (2004) investigated the effect of a 42-day administration of a resin extract of *Boswellia serrata* in dogs with osteoarthritis and spinal disease, demonstrating the potential of this plant extract to improve the clinical condition of dogs with osteoarthritis [214]. The anti-rheumatic and anti-inflammatory properties of this plant-based supplement stem from the presence of several compounds that interfere with the production or action of mediators involved in the inflammatory process, including TNF-α, IL-1β, NO, mitogen-activated protein kinases (MAPKs), gamma-interferon (IFN-γ), and IL-6 [217,218]. Boswellia extract was also associated with increased antioxidant defense and inhibition of lipid peroxidation, thus showing it possesses an overall antioxidant power [218]. However, although being natural, plant extracts are not completely harmless. For instance, the administration of a garlic extract to healthy adult mixed-breed dogs resulted in significantly decreased erythrocyte count, hematocrit, and hemoglobin concentration, along with Heinz bodies formation and increased erythrocytic methemoglobin concentration. Furthermore, morphological abnormalities, including the formation of eccentrocytes, was observed [219]. Moreover, side effects following the supplementation of plant extracts may also derive from the contamination of the material used during the extraction, including also the potential presence of heavy metals, fungi or chemical substances, such as pesticides [220].

### 3.4. Dietary Supplements

Dietary supplements are substances intended to be administered orally, either with food or separately, and that, despite being different from drugs, produce specific health benefits for the animals to which they are administered [40]. Multivitamins, chondroprotective agents, and glucosamine are among the most popular supplements among pet owners. However, minerals, amino acids, enzymes, organ tissues, and metabolites are also quite common [40,221]. In companion animals, they are often used to support joint health and gastrointestinal function, while also promoting cognitive function, skin and coat health, and the well-being of the cardiovascular and urinary systems [40].

#### 3.4.1. Health Benefits of Vitamins and Mechanisms of Action

Vitamins are small, organic, essential molecules that fulfill important roles in animal organisms at all stages of life. Based on their solubility, they are classified into fat-soluble (A, D, E, K) and water-soluble (B complex and C) vitamins [222,223]. Furthermore, the B complex group includes nine different vitamins, specifically thiamine (B1), Riboflavin (B2), Niacin (B3), Pantothenic acid (B5), Pyridoxine (B6), Biotin (B7), Folate (B9), and Cobalamin (B12) [223]. All of them play essential roles in the health and well-being of animals, thereby supporting their longevity, and their deficiency can lead to serious detrimental consequences.

For instance, vitamin A is involved in the morphogenesis of eyes, the retention of their structural integrity, and in ensuring the correct structure and function of retinal photoreceptors, thereby protecting vision. Furthermore, it has antioxidant and immunomodulating properties, plays a crucial role in cell growth and differentiation, which is particularly relevant during embryo development, and ensures the health of the reproductive tract [224].

Vitamins belonging to the B complex act as cofactors or coenzymes and are involved in the metabolism of carbohydrates, proteins, and lipids [225,226,227].

Vitamin C is known for its antioxidant properties, but it is also involved in tissue growth and maintenance, the modulation of the immune system, and the synthesis of vasoactive substances, including catecholamines and vasopressin [228].

Vitamin D is essential for calcium homeostasis and to ensure the correct development and maintenance of bones [229]. However, hypovitaminosis D has also been observed in extra-skeletal pathologic conditions, including gastrointestinal, renal, cardiac, infectious diseases, cancer, and inflammation. Therefore, research is now focusing on understanding whether vitamin D is somehow linked to the onset of these pathologies or if it can be used as a marker of illness [230].

Vitamin E, also known as alpha-tocopherol, has a potent free radical scavenging activity, thus protecting against oxidative stress. It also works synergistically along with other antioxidant molecules. For instance, vitamin C has been observed to support the regeneration of vitamin E during the process of free radical scavenging, and supplementation with a blend of vitamins B, C, and β-carotene has shown increased cell protection and reduced oxidative damage [231].

Finally, vitamin K is well known for its role in the coagulation cascade and is used in veterinary medicine to treat anticoagulant rodenticide toxicosis in both dogs and cats [232,233]. However, it also has antioxidant and anti-inflammatory activities, as well as protective effects on several districts, including the nervous, cardiovascular, and immune systems, as well as bones, and can reduce the incidence of certain pathological conditions, such as type 2 diabetes mellitus and pathogenic thrombosis [234].

Vitamins play a pivotal role in all animal organisms. However, their metabolism can differ among different species. For instance, dogs and cats are unable to synthesize vitamin B1 [225]. Moreover, compared to other species, they have physiological enzymatic deficiencies that limit their ability to synthesize vitamin D3 [230,235], and cats are unable to convert dietary β-carotene into vitamin A and to synthesize niacin from tryptophan [235].

Hence, vitamin supplementation becomes relevant primarily in cases where species-specific enzymatic limitations pair with suboptimal environmental or nutritional management, that might harm pets’ health and reduce life expectancy by impairing organ function and increasing oxidative and inflammatory damage. Nevertheless, vitamins should be supplemented cautiously, since excessive vitamin intake, especially concerning fat-soluble vitamins, can result in toxicosis, also known as “hypervitaminosis” [236]. Concerning hypervitaminosis A, dogs and cats appear to be more tolerant compared to other domesticated animals; however, they can develop vitamin A toxicosis, with symptoms including diarrhea, reduced appetite, neurological symptoms, bone demineralization, and reduction in thyroxin plasma concentration [224]. Hypervitaminosis D often follows excessive vitamin D dietary supplementation, rodenticide poisoning, or treatment with substances containing vitamin D2, D3, or vitamin D metabolites or analogues. It is one of the main causes of hypercalcemia both in dogs and cats, and symptoms include lethargy, inappetence, gastrointestinal disturbances, such as vomiting and melena, polyuria, and polydipsia [237,238]. Finally, while no hypervitaminosis K has been described, Vitamin E toxicosis is rare in companion animals; however, overdosing can result in bleeding problems due to vitamin K inhibition and impairment of leukocyte and lymphocyte functions [236].

#### 3.4.2. Health Benefits of Minerals and Mechanisms of Action

Minerals are essential micronutrients involved in several biological processes, ranging from initiating hormone production to tissue and subcellular functions [239].

According to the required daily amount, they are classified into two main groups. The “macro elements” include calcium, chloride, magnesium, phosphorus, potassium, and sodium, while the “trace minerals” include iron, zinc, selenium, copper, manganese, and iodine [4,239,240]. Mineral supplementation is essential for maintaining essential bodily processes. However, both excess and deficiency can result in serious health consequences, such as metabolic and neurological disorders, as well as musculoskeletal, cardiovascular, and oncological diseases, in both humans and animals [241,242].

For instance, zinc, copper, and manganese impact the antioxidant system by influencing the activity of superoxide dismutase (SOD) [243]. Zinc is involved in the proper functioning of alkaline phosphatases, and its optimal supplementation has been associated with increased longevity in dogs [244].

Moreover, mineral imbalance has been linked to the onset of several pathologies. For example, zinc deficiency has been associated with skin [245,246,247] and behavioral [248] disorders. Conversely, a high zinc serum concentration results in zinc toxicosis, characterized by inappetence, vomiting, and regenerative anemia [249]. Moreover, research conducted by Vitale et al. (2019) hypothesized that an imbalance of manganese, selenium, and zinc serum concentrations may play a role in canine idiopathic epilepsy [250].

Calcium and phosphorus play a crucial role in the health of dogs and cats. Beyond its involvement in bone development and skeletal health, calcium participates in a variety of processes, including blood coagulation, nerve impulse transmission, cardiac contractility, hormone and neurotransmitter secretion, enzyme reactions, and intestinal motility [251,252]. Phosphorus, on the other hand, contributes to the development of bones and teeth, the maintenance of osmotic and acid-base balance, electrolyte transport, and several enzymatic systems, as well as taking part in the synthesis of nucleic acids [253].

Imbalance in the concentrations of these elements, whether characterized by excess or deficiency, can lead to potentially life-threatening complications requiring emergency treatment and prolonged hospitalization [254,255,256,257]. Moreover, imbalanced levels of calcium and phosphorus, along with dysregulation of vitamin D and parathyroid hormone, can facilitate the onset of chronic kidney disease—mineral and bone disease (CKD-MBD) [258]. Also, selenium is fundamental in keeping humans and animals healthy. It is involved in several mechanisms, including redox balance, immune modulation, DNA synthesis, and ensuring the proper functioning of the reproductive system and thyroid metabolism [259,260,261]. Furthermore, research conducted on canine neoplastic cell cultures has enabled researchers to speculate on the potential role of selenium in cancer prevention [262,263,264].

The aforementioned examples highlight the pivotal role of minerals in maintaining the well-being of companion animals. Therefore, it is evident that ensuring proper mineral supplementation for pets can significantly impact their physical well-being and prevent the onset of potentially life-threatening conditions that could compromise their health and longevity. However, minerals should be supplemented carefully since an excessive intake can lead to severe toxicosis [265], resulting in a wide range of manifestations, including gastrointestinal symptoms, anemia, lethargy, anorexia, and emaciation [261,266,267].

#### 3.4.3. Health Benefits of Glutathione and Mechanisms of Action

Glutathione, also known as gamma-glutamyl-cysteinyl-glycine (GSH), is a tripeptide synthesized primarily in the liver from glutamate, cysteine, and glycine, and is widely recognized for its antioxidant properties [268]. It is synthesized in the cytosol and distributed to intracellular organelles, including mitochondria, which are the primary source of reactive oxygen species (ROS) [269,270]. Besides being the primary intracellular antioxidant agent, it is involved in several cellular functions, including the modulation of cellular proliferation, apoptosis, and gene transcription [271]. Moreover, it also plays a role in the detoxification of xenobiotics and endogenous compounds [269,272]. It exists in two primary forms, reduced (GSH) and oxidized (GSSG) glutathione, whose ratio is used to determine the redox status of the cells. The optimal GSH/GSSG ratio is >100, while a ratio of 1:10 is characteristic of cells exposed to oxidative stress (OS) [269].

GSH exerts its protective effect against cellular oxidative damage both directly and as a cofactor of antioxidant and detoxification enzymes, including glutathione peroxidases, glutathione S-transferases, and glyoxalases [273]. The direct antioxidant and scavenging abilities of GSH are attributable to its capacity to donate electrons, which enables GSH to neutralize ROS, including superoxide, hydroperoxyl, and hydroxyl radicals [274]. Hence, GSH is fundamental to preserving intracellular redox balance and mitigating OS [275].

Obstructing the OS is of utmost importance to ensure pets’ health and longevity, as it has been linked to the onset of a multitude of pathologies in both dogs and cats, such as gastrointestinal, hepatic, pancreatic, and endocrine diseases [259,276,277], and has been recognized as a contributor to aging [278]. All these conditions can influence the quality of life of companion animals and reduce their lifespan. Moreover, reduced GSH concentration has been detected in ill dogs and cats, which was also correlated with illness severity and increased mortality [279]. Particularly, low GSH values were found in both cats and dogs suffering from liver diseases [280]. This can be linked to the fact that the liver is the primary GSH producer [281] and contains the highest GSH concentration; hence, liver dysfunction can cause a reduction in GSH concentration, both due to reduced synthesis and increased use [282]. In human medicine, GSH supplementation is used for its beneficial effects on health, including anti-aging, skin-protective, immune-modulating, and hepatoprotective properties [283,284,285]. In veterinary medicine, glutathione-based supplements are less popular. However, some studies support its usefulness in supporting animal health. For instance, Vulcano et al. (2012) observed that GSH supplementation reduced acetaminophen (APAP)-induced methemoglobin formation and hepatotoxicity in cats, suggesting that GSH can be used as a therapeutic agent to mitigate APAP toxicity [286]. Glutathione supplementation can also be beneficial in dogs by increasing erythrocyte GSH levels and exerting hepatoprotective effects, resulting in improved liver parameters, notably alanine transaminase (ALT), alanine aminotransferase (AST), alkaline phosphatase (ALP), gamma-glutamyl transferase (GGT), and bilirubin (BIL) [287].

Besides being the most important antioxidant molecule of the organism, GSH also supports cellular functions and protects endogenous and exogenous toxic compounds, thereby contributing to pets’ health and longevity.

#### 3.4.4. Health Benefits of Glucosamine and Chondroitin Sulphate and Mechanisms of Action

Glucosamine and chondroitin sulphate (CS) are two nutraceutical substances primarily employed in veterinary medicine for the management of osteoarthritis (OA) due to their chondroprotective properties [288]. CS is a natural glycosaminoglycan that is detectable in all connective tissues, particularly in articular cartilage [289]. Its primary function is to ensure joint health; however, it also possesses antioxidant, anti-inflammatory, and immune-modulating properties [290]. Glucosamine, on the other hand, is an amino sugar that is abundant in articular cartilage, intervertebral discs, and synovial fluid. It can be isolated from the shell of shellfish [291] and is primarily employed orally in the management of OA [292].

CS and glucosamine act synergistically to promote the synthesis of glycosaminoglycan in chondrocytes. Moreover, CS was also associated with the inhibition of collagenase and aggrecanase, two proteolytic enzymes involved in the degenerative processes of cartilage. Overall, the combination of CS and glucosamine hinders cartilage degeneration, thereby positively impacting the progression of OA [293].

OA is a multifactorial joint pathology characterized by articular chronic inflammation and cartilage damage, including irregularity and focal erosions. These alterations gradually spread to the entire joint, resulting in pain and loss of function [294,295], which negatively impacts the quality of life of the affected animals.

OA represents the most commonly diagnosed joint pathology in veterinary medicine. Indeed, it is frequently observed in aging dogs, particularly in large and giant breeds, as well as in cats [295,296,297]. Moreover, OA is recognized as the leading cause of chronic pain [298], which can result in premature euthanasia of companion animals if under-recognized or under-managed [299], thus negatively impacting animals’ longevity.

To date, the use of both CS and glucosamine in the management of OA is controversial, as research conducted has shown heterogeneous results [288,292,300]. Nevertheless, they can mitigate OA damage, thus improving pets’ quality of life. In a recent in vitro study, Bai et al. (2024) observed the ability of CS to suppress the secretion of pro-inflammatory cytokines in lipopolysaccharide (LPS)-challenged canine and feline chondrocytes [301]. Moreover, it also promoted cell viability and proliferation, confirming its ability to modulate inflammation and its potential role in the treatment of OA. Furthermore, in vivo research has highlighted the efficacy of combining glucosamine and chondroitin sulfate in reducing joint pain and their chondroprotective effects in rat models [302,303]. Accordingly, oral administration of a combination of CS and glucosamine in dogs with OA resulted in clinical amelioration and improvements in pain scores [304]. However, results are conflicting, and more research is needed to assess the fundamental role of CS and glucosamine in the management of OA in companion animals [305,306,307,308,309].

Altogether, the vast array of nutraceutical substances, from omega-3 fatty acids to pre- and probiotics, plant extracts, vitamins, minerals, and chondroprotective agents, suggests their pivotal role in promoting systemic health, preventing disease, and ultimately supporting the longevity and quality of life of companion animals.

### 3.5. GBA and Gut Health Modulation by Nutraceuticals

Increasing evidence suggests that gut microbiota balance and stability, plays a crucial role in maintaining the host’s health and aging-related changes, thus contributing to the human and animal longevity [310,311,312]. Conversely, dysbiosis can lead to the onset of pathological conditions, including mental disorders and anxiety, that negatively influence the pet-owner relationship, potentially leading to abandonment or euthanasia [313].

The gut exerts a dynamic interaction with other organs (liver [314], kidneys [315], lungs [316], and brain [317,318]) through several pathways, referred to as “axes”, positively or negatively modulating their function [319]. Particularly, the GBA is a complex, bidirectional communication system between gut microbiome and the central nervous system (CNS) [320,321], whose interplay is so intimate that the gut is often referred to as a “second brain” [322].

The gut microbiome produces multiple metabolites, neuroactive substances and hormones that can reach the brain through the enteric nervous system (ENS) [323], whose mediator is GABA, the vagus nerve, circulation, or the immune system and modulate its function [324]. On the other hand, the brain can communicate with the gut microbiota by inducing neurons, immune cells, and enterochromaffin cells to secrete signaling molecules, thereby modulating gut functionality and environment (pH, motility, and mucus), and creating optimal conditions for the microflora [325].

The gut microbiota can also influence the production of neurotransmitters such as dopamine (DA), serotonin (5-HT), glutamate, and gamma-aminobutyric acid (GABA), which are involved in brain function, cognition, and social behavior [326,327,328].

Serotonin, for instance, was shown to play a role in circadian rhythms and multiple psychological processes, including mood, perception, reward, anger, aggression, fear, memory, sexuality, addiction, and attention [329]. Conversely, dopamine can regulate various functions, including cognition, emotion, positive reinforcement/reward-driven learning, food intake, and motivation [330,331].

The GBA is a relatively novel concept that has garnered the interest of researchers due to its health-enhancing potential, including a possible role in maintaining homeostasis and modulating behavior [332], anxiety, depression, affect, motivation, and cognitive functions [333]. Particularly, dysregulation of GBA crosstalk has been associated with many pathological conditions, including metabolic syndrome, psychiatric disorders such as depression and anxiety, and autism spectrum disorders (ASD). GBA crosstalk has also been linked to neurodegenerative diseases, such as Parkinson’s disease (PD) and Alzheimer’s disease (AD) [334]. In addition, research has shown that the gut microbiota of people suffering from mental disorders, including anxiety, is altered compared to healthy controls [335,336,337,338].

Similarly to humans, the existence of the gut–brain axis and the link between gut microbiota alterations and pathological conditions has been documented in companion animals [313]. Likewise, in pets, dysbiosis has been linked not only to neurological diseases, such as idiopathic epilepsy [339,340], but also to gastrointestinal [341,342] and metabolic diseases [341], exocrine pancreatic insufficiency [343], behavioral disorders [196,197,198], and cancer [344].

To ensure GBA health, thereby reducing aging-related changes and possibly promoting longevity, a correct nutrition based on nutraceutical substances and specific ingredients that can positively influence the composition of the gut microbiota is mandatory [345].

Nutraceutical substances, including “biotics” (pre-, pro-, post-, and synbiotics), as well as natural ingredients and extracts, have been shown to support gastrointestinal function and positively modulate gut microbiota composition [346,347,348,349,350,351].

Healthy dogs supplemented with oat groats, beet pulps, and pea fibers, prebiotics (i.e., inulin), probiotics, and immune supporters (i.e., spray-dried animal plasma and yeast-derived fermentation products), improved gastrointestinal and immune health, stool quality, and fecal metabolites [346]. Notably, a reduction in butyrate, isobutyrate, isovalerate, total branched-chain fatty acids (BCFA), indole, and ammonia fecal concentrations, and an increase in fecal 7-methylindole and calprotectin were observed. Similarly, the supplementation of *Saccharomyces cerevisiae* fermentation products positively shifted the gut microbiota composition in adult dogs, by increasing fecal *Bifidobacterium* and decreasing *Fusobacterium*, phenol, and indole concentrations [347]. Moreover, this prebiotic also enhanced the T helper 1 lymphocyte (Th_1_) response and decreased toll-like receptor (TLR) responsiveness, as well as the secretion of TNF-α and pro-inflammatory cytokines, demonstrating its potential anti-inflammatory and immune-modulating activity. Positive effects on gastrointestinal function and gut microbiota were also observed from the supplementation of other prebiotics, including miscanthus grass fiber [348], red ginseng dietary fiber [349], bioactive peptides [350], and *Saccharomyces cerevisiae* cell wall [351]. The latter, in particular, was responsible for a reduction in *C. perfringens* concentration in gut microbiota, thereby indirectly improving lactic acid concentration, which suggests an enhanced proliferation of beneficial lactic acid bacteria [351]. Prebiotics and probiotics also exert a beneficial effect on canine intestinal health by modulating the microflora through a direct competition for nutrients or the production of substances, such as bacteriocins, which inhibit the proliferation of other species, especially pathogens [352]. For example, the administration of Kefir, a fermented dairy product rich in lactic acid bacteria and beneficial yeasts, it has been shown to significantly reduce *Clostridiaceae*, *Fusobacteriaceae*, and *Ruminococcaceae* in healthy adult dogs while increasing *Prevotellacea*, *Selenomonadaceae*, *Sutterellaceae*, and lactic acid bacteria, thereby emerging as a novel functional pet food [353]. Finally, a favorable modulation of intestinal microbiota was attained in healthy dogs that were administered a snack containing 0.5% krill oil (KO). KO facilitated the proliferation of beneficial bacteria, including *Bifidobacterium*, *Muribaculaceae*, *Ruminococcaceae*, *Faecalibacterium*, and *Prevotellaceae*, thereby exerting a positive influence on the overall health of the canines [354].

Besides influencing gut microbiota composition, nutraceuticals can promote health and longevity by playing a role in the management of gastrointestinal diseases, accelerating the healing process, and the animals’ recovery. For example, a probiotic supplement containing four different *Lactobacillus* strains enhanced the multiplication of beneficial gut microbes to the detriment of potentially pathogenic ones, thereby promoting recovery in puppies with gastroenteritis [355]. Likewise, probiotic supplementation restored normobiosis in adult dogs suffering from diarrhea, thereby accelerating their recovery [356,357].

Dogs supplemented with a probiotic containing seven bacterial strains belonging to the genera *Lactobacillus*, *Bifidobacterium*, and *Streptococcus* showed faster recovery and accelerated normalization of the gut microflora compared to placebo control [192]. Similarly, supplementing dogs suffering from acute or intermittent diarrhea with a specific probiotic combination of Lactobacilli resulted in decreased concentrations of *Clostridium perfringens* and *Enterococcus faecium* along with normalization of stool consistency and improved well-being [358].

Currently, research is focusing on molecules derived from plants, such as turmeric, *Boswellia serrata*, or Aloe vera, whose bioactive substances, including curcumin, boswellic acid, vitamins, terpenoids, and flavonoids, have been proposed in the management of gastrointestinal disorders, such as colitis and IBD, through their anti-inflammatory and antioxidant properties. However, further research is necessary, particularly in the context of cats, to fully understand the potential of these substances [359].

### 3.6. Behavioral Disturbances and Cognitive Impairment Management by Nutraceuticals

Companion animals’ behavioral issues are a significant concern in the relationship between owners and pets [360]. Sharing everyday life with a pet that exhibits behavioral problems, such as aggression, destructive behaviors, vocalizations, or elimination issues, can cause stress in owners, undermine their overall well-being and social interactions with neighbors, as well as lead to serious consequences for the pet, including abandonment [361,362]. The onset of stress and behavioral disorders can be attributed to a variety of factors, including an imbalance of neurotransmitters like GABA, serotonin, dopamine, melatonin, histamine, acetylcholine, and norepinephrine [328,363]. These molecules have been linked to a range of neuropsychological processes, including anger, aggression, anxiety, motivation, reward, and emotional behaviors [328,364,365]. Consequently, an imbalance in their concentration has the potential to induce behavioral issues, as observed in dogs [366,367,368,369].

Growing evidence is supporting the relationship between gut microbiota and the behavior of companion animals through the gut–brain axis [196,360,370], possibly due to the activity of Lactobacilli, Bifidobacteria, and *Escherichia coli*, able to produce specific molecules, including neurotransmitters, which impact behavior and neurodevelopment [257,363].

In this sense, nutritional interventions have been proposed as a potential strategy to mitigate undesirable behaviors and enhance the quality of shared living [196].

Nutraceuticals, in particular probiotics [199,200], along with novel techniques, such as fecal microbiota transplantation (FMT), might represent a sustainable way to modulate gut microbiota and influence the GBA and, indirectly, pets’ behavior [371]. For example, *Lactobacillus plantarum* PS128 has been shown to improve general emotional stability in dogs while reducing the severity of behavioral problems, including aggression, separation anxiety, and compulsive disorders [199]. The dogs’ emotional and cognitive status, as well as behavioral issues and severity, were evaluated using the Evaluation of Dogs’ Emotional and Cognitive Disorders (EDED) scale and the Canine Behavioral Checklist (CBD) scale, respectively. Both scales’ values resulted decreased, thereby confirming the favorable impact of the probiotic on the animals’ behavior. In addition, a significant decrease in the serotonin turnover ratio (5-HIAA/5-HT) was observed in dogs suffering from separation anxiety, which was associated with a slowdown of serotonin metabolism after the PS128 supplementation.

Similarly, *Lactobacillus plantarum* LP185^TM^ was observed to reduce aggression and anxiety, as indicated by the Canine Behavioral Assessment & Research Questionnaires (C-BARQ) [200]. The probiotic supplementation resulted in a decrease in the time required for the subjects to settle after the owner departure and in the dog daytime activity, and an improvement in their sleep patterns. These results suggest that supplementation with *Lactobacillus plantarum* LP185^TM^ may be a promising strategy for treating behavioral issues.

Also, vegetal substances and extracts have been shown to modulate companion animals’ behavior. For instance, a 45-day administration of a nutraceutical diet containing *Punica granatum*, *Valeriana officinalis*, *Rosmarinus officinalis*, *Tilia* species, *Crataegus oxyacantha*, green tea extract, L-tryptophan, and an omega-3/6 in a ratio of 1:0.8 resulted effective in the management of behavioral issues (anxiety and chronic stress) in dogs [48]. Specifically, the animals receiving the nutraceutical diet showed increased plasma concentrations of serotonin, dopamine, and β-endorphins, together with decreased concentrations of noradrenaline and cortisol compared to the control group. Likewise, a similar nutraceutical supplementation to dogs with evident symptoms of behavioral disturbances was also linked to the improvement of conduct, such as anxiety, diffidence, irregular biorhythm, reactivity, irritability, and alertness, and their related clinical signs, including cutaneous and gastrointestinal signs [47]. The supplementation of a nutraceutical product containing anti-inflammatory compounds, pre- and probiotics, 5-hydroxytryptophan, and L-theanine alleviated stress and anxious behaviors in dogs, thus demonstrating the connection between nutraceuticals, microbiota, and behavior [372]. Finally, 20 healthy dogs receiving *Melissa officinalis* hydro-alcoholic extract showed a decrease in plasmatic 4-hydroxybutyric acid (GHB), one of the main metabolites synthesized from GABA [373]. The reduction was ascribed to the inhibitory activity of certain compounds present in the natural extract on GABA transaminase, which led to the accumulation of GABA in the brain and the manifestation of calming effect.

Behavioral disorders, such as disorientation, altered interactions with humans and other pets, and sleep–wake cycle disturbances, have also been observed in conjunction with cognitive impairment, in particular in elderly pets [374,375]. In this case, nutraceutical supplementation with *Grifola frondosa*, *Curcuma longa*, *Carica papaya*, *Punica granatum*, *Aloe vera*, *Polygonum cuspidatum*, *Solanum lycopersicum*, *Vitis vinifera*, *Rosmarinus officinalis* and an Omega 3/6 ratio of 1 : 0.8 showed to counteract the onset of cognitive impairment by increasing serum concentrations of brain-derived neurotrophic factor (BDNF), which is involved in neuroprotection [376].

Furthermore, the administration of Ginkgo biloba leaf extracts has been shown to ameliorate most of clinical indications associated with cognitive impairment, including disorientation, sleep disturbances, behavioral changes, and general physical condition in elderly dogs, suggesting its potential in enhancing their quality of life [377]. Also, nutraceutical supplements have been shown to exert beneficial effects on memory [378] and in the prevention of cognitive decline [379] in aged dogs. In cats, nutraceutical substances, such as fish oil, vitamins, antioxidants, minerals, botanicals, and amino acids, can help mitigate mild cognitive dysfunction syndrome (CDS) and enhance longevity [380].

It appears evident, then, that the evidence summarized here underscores the potential of nutraceuticals as a sustainable and effective strategy for the management of behavioral disorders and cognitive impairment in companion animals, ultimately supporting improvements in their welfare and longevity.

### 3.7. Joint Health Management by Nutraceuticals

Advancements in veterinary medicine and animal nutrition have contributed to an increase in the lifespan of companion animals [81]. Consequently, a greater number of animals are reaching advanced age and, consequently, developing age-related conditions, in particular OA [381], a widespread, degenerative, and inflammatory musculoskeletal disorder [382,383,384]. OA is characterized by a deterioration of the normal structure of the joint, which includes the degradation of articular cartilage, the remodeling of bone, the formation of osteophytes, subchondral bone sclerosis, chronic synovitis, and pain [214], which strongly reduces the quality of life of the affected animals [385].

In cats, OA may manifest with slight behavioral changes indicative of pain, rather than the conventional clinical signs observed in dogs (pain or tenderness, decreased range of motion, swelling, stiffness, muscle atrophy, crepitus, and effusion) [384]. A primary concern associated with OA is its incurability [214,383]. Consequently, treatment regimens should prioritize the mitigation of its progression, the reduction in pain, the improvement of motor function, and the enhancement of the quality of life for affected pets [214,383].

Although the presence of different approaches to managing this condition, including pharmacological [e.g., opioids or non-steroidal anti-inflammatory drugs (NSAIDs)] [386], dietary modifications and surgery [384,387], NSAIDs are the primary therapeutic choice.

However, the prolonged use of NSAIDs has been associated with adverse effects on various organs, including gastrointestinal erosion or ulceration, hepatic and renal damage, accelerated cartilage degeneration, and delayed bone healing [386,388]. For this reason, research has centered on identifying novel therapeutic options, such as nutraceuticals, which might constitute a more natural and safer alternative to NSAIDs [386]. Omega-3 fatty acids, glucosamine, chondroitin sulfate, collagen derivatives, green-lipped mussel, and various herbal medicines, including *Boswellia serrata*, were shown to enhance joint health and alleviate pain in dogs afflicted with OA [214,384,389,390]. For example, supplementing dogs with omega-3 fatty acids improved their joint conditions, reducing lameness and pain and improving their ability to rise from a resting position, walk, and play [141,391,392,393,394]. According to a study of Mehler et al. (2015), a daily administration of EPA and DHA in dogs suffering from OA significantly improved crepitus, pain, effusion, muscle atrophy and chronic nerve stimulation after 84 days [388]. Based on these observations, Corbee et al. (2013) found that 10 weeks of omega-3 fatty acids supplementation improved the behavior and locomotion of cats with OA [395]. Consequently, their activity level, movement, and interaction with their owners increased, and stiffness during gait decreased.

Furthermore, according to Fritsch et al. (2010), the supplementation of an omega-3-enriched diet in dogs suffering from OA and receiving the NSAID carprofen led to a reduction in its usage [140]. However, it should be noted that this study involved different veterinarians who adopted their own criteria to modulate the carprofen dosage. Therefore, this may have influenced the study’s findings. Other nutraceutical products proposed as potential treatments or adjuvants for OA include collagen and its derivatives, glucosamine, and plants or plant-derived supplements, such as *Boswellia serrata*, curcuminoids, and cannabidiol [214,396,397,398,399,400,401]. Recent findings have indicated that supplementing dogs with collagen hydrolysate or sulfated glucosamine, in comparison to a control diet, has led to a more significant reduction in lameness, OA-related symptoms, and pain.

Both of these nutraceuticals have been shown to enhance mobility, agility, and overall quality of life [396]. These outcomes were associated with the capacity of collagen hydrolysates to decrease the blood concentration of matrix metalloproteinase 3 (MMP-3), a proteolytic enzyme implicated in cartilage degradation [402], without affecting the levels of its inhibitor (Tissue Inhibitors of Metalloproteinases-1—TIMP-1). Moreover, the influence of both compounds on multiple biochemical processes has been postulated, with the potential to promote cartilage health [396]. This hypothesis suggests a positive impact on joint health, thereby substantiating their application in the management of OA.

In addition, beneficial effects on joint health have also been reported for collagen peptides [403,404]. In a study of dogs with OA, the administration of bioactive collagen peptides (BCP) via oral supplementation for 12 weeks yielded to superior outcomes in terms of alleviating the symptoms associated with OA when compared to other nutraceuticals (i.e., omega-3 fatty acids and Vitamin E) [398]. An improvement in clinical symptoms was also observed after oral supplementation with undenatured type II collagen (UC-II ^®^) in subjects suffering from OA [397]. In this study, metabolic alterations in the synovial fluid composition of joints in dogs suffering from OA were observed and compared to those in healthy controls. The alterations included increased levels of β-hydroxybutyrate, glutamine, trimethylamine-*N*-oxide (TMAO), creatine/creatinine, alanine, and histidine. The oral administration of UC-II resulted in a rebalancing of metabolism within the joint, as evidenced by the absence of β-hydroxybutyrate, a characteristic compound identified in the joints of dogs afflicted with OA.

As for plants and plant-derived substances, a substantial body of research has been conducted on their effects on OA, alone or in combination with other nutraceuticals [214,399,401,405,406]. *Boswellia serrata* is one of the most common nutraceuticals among those who support joint health. *Boswellia*-based products have produced positive results in various formulations. For example, a *Boswellia serrata* resin extract was shown to reduce OA symptoms in dogs that were not treated with any other anti-inflammatory agent after six weeks from the first day of administration [214]. Furthermore, the combination of *Boswellia serrata* and UC-II^®^ improved mobility and synovial metabolomic composition in sick dogs’ joints after four weeks of administration, like the previous study [405]. In addition, *Boswellia serrata* is often an ingredient in nutraceutical blends that also contain other natural substances, such as chlorophyll, green tea extract, and natural chondroprotectants [406]. These blends may also contain fatty acids, whole freeze-dried green-lipped mussel powder (*Perna canaliculus*), and devil’s claw (*Harpagophytum procumbens*) [399]. These blends have been seen to reduce pain and clinical signs of OA and slow down the progression of the disease.

Although literature on this topic is extensive and opinions are sometimes conflicting, the reported evidence suggests that integrating nutraceuticals into multimodal management strategies is a scientifically substantiated, physiologically targeted approach to supporting joint integrity, enhancing longevity, and improving quality of life in companion animals.

### 3.8. Skin and Coat Management by Nutraceuticals

Skin is the largest organ of the body in mammals and represents their first line of defense [407]. It is strictly connected with the coat, both contributing to many fundamental functions, such as physical protection, thermoregulation, production of substances, including Vitamin D, and sensory perception [65,408]. The coat can also be considered a mirror of the overall health status, quality of life, physical and nutritional conditions of cats and dogs [409]. Ensuring an optimal healthy status of skin and coat is thereby pivotal to keeping their functions. Indeed, hair loss exposes skin to environmental stressors and may lead to the onset of diseases, such as dermatosis, thereby weakening its protective function.

Nutritional deficiencies have been shown to result in skin disorders, including seborrhea, keratinization, erythema, poor hair growth or alopecia, and greasy skin, which can facilitate the onset of pruritus and bacterial infections [410], thus potentially affecting the integrity of skin.

On the other hand, multiple nutraceuticals, including fatty acids [411,412], botanicals [42], probiotics [413,414], and trace minerals [415] can positively impact the skin and coat in both physiological and pathological conditions [409]. An increase in the total PUFAs content of a diet, from 9 to 13%, was shown to significantly enhance skin and coat quality, especially glossiness and softness of healthy dogs after 12 weeks supplementation, if compared to a control diet richer in saturated fatty acids [416].

The health status of the skin and coat also benefited from the administration of capsules containing EPA, DHA, and vitamin E. The observed benefits reached their zenith two months after the beginning of the supplementation and persisted for up to one month following its withdrawal [411].

KO constitutes an additional natural source of EPA and DHA. In a recent study conducted by Wang et al. (2025), the administration of a dietary supplement containing KO for 8 weeks in healthy dogs resulted in augmentation of total amino acids, particularly methionine, in the air of the supplemented subjects [354]. Furthermore, a decrease in thickness accompanied by an increase in hair softness was observed, thereby substantiating the capacity of KO to enhance the overall quality of canine hair.

The presence of trace minerals is of critical importance to the well-being and aesthetic quality of animals. Indeed, in a recent study by Amundson et al. (2025) the supplementation with zinc (Zn), manganese (Mn), copper (Cu), and iron (Fe) improved the hair quality, growth, and shedding, with more evident results in the dogs receiving acid-complexed organic trace mineral sources than in those receiving the standard inorganic source [415].

Probiotics, as well, have been observed to contribute to improving hair conditions in 5 healthy cats that received a probiotic supplement consisting of *Bifidobacterium lactis* and *Lactobacillus plantarum* for 28 days and were weekly assessed for body weight, overall physical condition, hair, and fecal [416]. Regarding hair quality, a statistically significant improvement was noted after 28 days of probiotic supplementation.

Supplementing a diet consisting of a blend of botanicals, zinc, and an omega-3/6 ratio of 1:0.8 to a dog with hind paw melanoma has been shown to reduce the lesion dimension and enhance the hematochemical profile and overall quality of life, thereby extending its median survival time [34]. Similarly, nutraceuticals exerted a favorable impact on a canine patient suffering from granulomatous dermatitis and chronic bilateral otitis. The dog was administered two nutraceutical diets for one month each. The first formulation, composed of fish proteins and potato carbohydrates, *Rosa canina*, *Salvia officinalis*, and *Vaccinium macrocarpon*, was tailored for cutaneous manifestations and resulted in a substantial decrease in the lesions associated with granulomatous dermatitis (i.e., papules, nodules, and plaques). Similarly, the second nutraceutical diet, based on fish proteins, rice carbohydrates, *Melaleuca alternifolia*, *Tilia platyphyllos scapoli*, and *Tilia cordata*, *Allium sativum*, *Rosa canina*, and zinc, was specific for auricular diseases and resulted in reduced inflammation, edema, erythema, and occlusion of the ear canal [55]. Similar results also emerged in a study conducted by Di Cerbo et al. (2016), where combining topical pharmacological treatment with nutraceutical supplementation for 90 days resulted in relieving the symptoms of chronic otitis externa, thereby improving the quality of life of these pets [46].

Nutraceuticals supplementation can result useful also in the management of allergic conditions in both cats and dogs. Gut microbiota modulation through probiotic administration, such as *Lactobacillus sakei* Probio-65, *Bifidobacterium bifidum*, *Lactobacillus acidophilus*, and *Enterococcus faecium*, improved all the symptoms related to canine atopic dermatitis (CAD), demonstrating the existence of a gut-skin axis [413,414,417]. Likewise, cats suffering from non–flea hypersensitivity dermatitis (NFHD), who were treated with methylprednisolone, exhibited reduced pruritus and a considerably prolonged time-to-relapse when receiving a co- and post-administration with the naturally occurring fatty acid palmitoylethanolamide compared to placebo [412].

In addition, the efficacy of nutraceuticals in mitigating the clinical symptoms and the severity of skin lesions in cats with cutaneous adverse food reaction (CAFR) has been well-documented [42,418,419,420]. CAFR is a group of reactions that can result from both allergies and intolerances. The administration of a nutraceutical diet based on *Aloe vera*, *Arctium lappa*, *Malva sylvestris*, *Ribes nigrum*, *Allium sativum* and Omega3/6 fatty acids (1:3 ratio), vitamin A, vitamin E, choline chloride, zinc sulphate monohydrate, cupric chelate glycine hydrate, and DL-methionine led to the restoration of physiologic dermal homeostasis, a reduction in the severity of the skin lesions, and an improvement in the clinical symptoms of CAFR, including drooling, pruritus, neck eczema, chronic conjunctivitis, and stomatitis [42].

In conclusion, these studies suggest the possible role of nutraceuticals in preserving the health and integrity of the skin and coat, thereby enhancing the quality of life and longevity in companion animals.

### 3.9. Immune Modulation by Nutraceuticals

As early as 1976, Scrimshaw hypothesized a link between nutrition and immune status [421]. Subsequent research has validated this association, and a great number of studies has been continuously increasing [422]. An imbalance or inadequate nutrition has a detrimental effect on immune function, increasing susceptibility to infections and compromising the organism’s capacity to repair damaged tissues or impeding the proliferation of malignant neoplastic cells [423]. In contrast, a favorable nutritional status, characterized by adequate intake of nutraceutical, particularly vitamins, trace elements, and omega-3 fatty acids, can support immune system function and positively impact health and longevity [424,425].

Particularly, supplementing healthy dogs with Chenpi powder (CPP) enhanced antioxidant and immunological functions [426]. Chenpi, the dry peel of *Citrus reticulata* cv. *Chachiensis*, contains a variety of beneficial compounds, including flavonoids and essential oils, which possess antioxidant, anti-inflammatory, and immune-modulating properties. The administration of CPP led to an enhancement in the activity of antioxidant enzymes, notably SOD, glutathione peroxidase (GSH-Px), and catalase (CAT), accompanied by a decline in serum malondialdehyde (MDA) concentration. Furthermore, a decrease in pro-inflammatory cytokines, such as IL-8 and TNF-α, and an increase in fecal secretory immunoglobulin A (SIgA) were observed. Similarly, an increase in SOD, GSH-Px, CAT, and Immunoglobulin G (IgG) serum content, along with a decrease in TNF-α, IL-8, and IL-1β was obtained after supplementing healthy dogs with KO [354], while oral supplementation of beta-glucans was shown to modulate the humoral immune response in healthy dogs, before and after vaccination [427]. Moreover, despite the absence of a significant impact on the white blood cell (WBC) count, the administration of dietary oat beta-glucans has been hypothesized to be implicated in the reduction in IL-4 serum concentration. This finding suggests a plausible mechanism through which these beta-glucans may hinder a T helper 2 lymphocyte (Th2) response following vaccination with the evaluated vaccine [428].

Also prebiotics, including GOS alone or in combination with manannoligosaccharides (MOS), FOS, and beta-glucans, enhanced immune function by increasing the number of polymorphonuclear cells, the phagocytosis index, and the ROS production for both Gram-positive and Gram-negative bacteria stimuli [429]. Similar results were also observed in beagle dogs after castration following the oral administration of a polysaccharide (from *Astragalus membranaceus (APS)*. Besides positively affecting wound healing, the administration of APS resulted in a reduction in pro-inflammatory cytokines IL-1β and TNF-α, as well as C-reactive protein, a marker of inflammation, in plasma [430]. Concurrently, APS increased the serum content of IL-10.

Immunomodulatory properties were also associated with the supplementation of *Ganoderma lucidum* (15 mg/kg bw), which increased vaccine-specific IgG against rabies in serum, enhance the phagocytic activity of macrophages, and the percentage of major histocompatibility II (MHC-II) from B cells [431].

Another study reported that supplementation with *Saccharomyces cerevisiae* fermentation products favorably influenced Th_1_ response and diminished TLR responses, consequently leading to a reduction in inflammation in adult dogs [347]. Conversely, the administration of *Saccharomyces cerevisiae* cell wall to cats did not exert any influence on the immune system [351]. On the other hand, cats supplemented with diets containing yeast-derived nucleotides, salmon oil, or L-arginine improved immune system functionality. This was evidenced by enhanced lymphocyte proliferative responses to the T-cell mitogen phytohaemagglutinin and increased blood leukocyte phagocytic activity [432]. Finally, in cats, the simultaneous supplementation of *Bifidobacterium lactis* and *Lactobacillus plantarum* enhanced mucosal immunity by increasing IgA serum concentrations and improving antioxidant defense, specifically CAT, SOD, and GSH-PX. Moreover, these probiotics also showed to provide anti-inflammatory benefits by modulating cytokines involved in inflammation, such as TNF-α, IFN-γ, IL-4, and IL-2 [417]. Similarly, supplementing healthy dogs with a multi-strain probiotic resulted in beneficial modulation of fecal microbiota by reducing harmful bacteria, such as *Clostridium perfringens*, while increasing beneficial Bifidobacteria and Lactobacilli. An increase in fecal IgA and plasma IgG was also registered in the treated group [433].

The immune-modulating properties of nutraceuticals have also been assessed in the management of immune-mediated pathologies. An in vitro study conducted in 2016 showed the ability of several botanicals (*Ascophyllum nodosum*, *Cucumis melo*, *Carica papaya*, *Aloe vera*, *Haematococcus pluvialis*, *Curcuma longa*, *Camellia sinensis*, *Punica granatum*, *Piper nigrum*, *Polygonum cuspidatum*, *Echinacea purpurea*, *Grifola frondosa*, and *Glycine max* to modulate the production of proinflammatory cytokines, such as IFN-γ, in both human and canine peripheral blood mononucleated cells (PBMCs) [434]. Moreover, *Haematococcus pluvialis*, *Glicine max*, and the mixture of all the 13 botanicals hindered oxytetracycline (OTC)-induced toxicity. This is of relevance since OTC toxicity has been linked to detrimental effects on the immune system and the onset of immune-mediated diseases [435,436,437]. The same nutraceuticals, combined with conventional pharmacological treatment, resulted beneficial in the management of keratoconjunctivitis sicca (KCS), a multifactorial ocular pathology resulting from an immune dysregulation that leads to inflammatory alterations of the lacrimal gland and the subsequent deficiency in the tear aqueous fraction [44]. Besides ameliorating the clinical symptoms, such as blepharospasm, ocular hyperemia, periocular swelling, and ocular discharge, the combination reduced also conjunctival inflammation, corneal keratinization, corneal pigment density, mucus discharge, and tear production. Likewise, the same combination of nutraceuticals and conventional pharmacological treatment significantly improved the clinical conditions of dogs suffering from epiphora by modulating tear overflow, conjunctival inflammation, corneal keratinization, and blepharitis [45].

The immune-modulating activity of the aforementioned nutraceutical resulted also effective in the management of naturally infected dogs with *Leishmania infantum* [52]. The administration of nutraceutical supplements led to a restoration of platelet number and CD3+ CD4+ Foxp3+ Regulatory T (Treg) cells population, as well as an improvement in the CD4/CD8 ratio. Furthermore, the dogs receiving the nutraceutical diet exhibited a progressive decline in CD3+ CD4+ IFN-γ + Th_1_ cells, reaching levels comparable to those observed in the healthy control group by the end of the trial. Positive effects in the management of leishmaniosis were also associated with the oral administration of a supplement containing nucleotides and active hexose correlated compound (AHCC), which has been shown to possess immune-modulating properties [438]. Clinically healthy dogs infected with *Leishmania infantum* were treated with this nutraceutical supplement for 1 year. The treatment did not exert significant changes between the supplemented group and the placebo group in terms of CD4+ and CD8+ levels, CD4+/CD8+ ratio, cytokine levels, protein electrophoresis, complete blood count, biochemistry, parasite load, or chronic kidney disease staging. However, the ELISA test showed a significant reduction in serological titers of antibodies against *Leishmania infantum*, proving its ability to prevent disease progression, thereby protecting animals from becoming sick.

Given the sensitivity of immune cells to changes in the balance between oxidant and antioxidant species, another important factor to ensure optimal immune function is to maintain optimal antioxidant levels [439]. In this context, the administration of a natural antioxidant blend containing S-acetyl-glutathione (SAG), a precursor of glutathione originating from the fermentation of *Saccharomyces cerevisiae*, resulted in an increase in the glutathione peroxidase levels, a key enzyme against oxidative damage [440]. A summary of the action mechanism, the clinical outcome, the animal model and the contribution to longevity exerted by omega-3 fatty acids, prebiotics and probiotics, plant extracts and dietary supplements is provided in Table 1.

## 4. Conclusions

The shift in societal perception of pets as integral members of the family unit heightened the interest in their psychophysical health, well-being, and possibly longevity. Moreover, pet–owner interaction has been recognized as a mutually beneficial endeavor, where owner derives physical and psychological benefits and pet receives sustenance and protection. In addition, the love and caring provided both ways can contribute to the enhancement of their respective quality of life. Consequently, social interactions function as a preventative measure against the development of stress-related pathologies, which are frequently related to behavioral disturbs in both species.

The aforementioned shift is also concomitant with the greater sensitivity to the quality of food, nutritional requirements and habits that owners provide to their “new family members”. In this context, nutraceuticals can improve pet–owner relationships through a direct modulation of GBA, thus helpful in the prevention or management of behavioral disturbs.

Besides modulating the GBA, by regulating the production of neuromodulators, nutraceuticals can also positively impact the health of joints, skin, coat, and the immune system, thus representing a fundamental tool to ensure the well-being of pets, improve their quality of life, and possibly promote their longevity.

## Figures and Tables

**Table 1:** Nutraceutical substances effects in dogs and cats.

Nutraceutical Substance	Mechanism of Action	Clinical Outcome	Animal Model	Contribution to Longevity
Omega-3 Fatty Acids	Anti-inflammatory and immune-modulating properties (IL-6, TNF-α, and eicosanoids reduction; impairment of leukocyte adhesion to endothelium, production of specific metabolites, competition with AA) [133,134,135,136,137,138,139,142,143,144] Cardiovascular function improvement (modulation of ionic currents in cardiac cells, reduction in leukocytic infiltration in ischemic tissue) [146,147,148] Positive influence on reproduction (modulation of molecules involved in reproduction, influence on sperm, oocytes, and corpus luteum development and functionality, improvement of semen quality) [149,150,151] Renal protection (modulation of cholesterolemia, triglyceridemia, GFR, oxidative damage, inflammation) [152,153] Neuroprotective and synaptogenesis promotion (support of brain development and, neurogenesis and glucose transport enhancement) [154,155,156]	Mobility improvement, pain relief and NSAID need reduction [140] Skin and coat quality improvement [354] Cardiovascular and renal health promotion [146,147,148] Reproductive functions and semen quality enhancement [49,151] Cognitive and vision support [154,155,156] Joint conditions improvement and OA-related symptoms reduction [246,391,392,393,394] Drooling, back and neck intense itching, neck eczema, chronic conjunctivitis and stomatitis improvement [42]	Dogs and cats with OA [246,391,392,393,394] Dogs with OA [140] Healthy dogs [354] Cats with CAFR [42] Dogs with ischemia-induced fatal cardiac ventricular arrhythmias [146] dogs suffering from infertility associated with hypospermia [49]	Diseases prevention and chronic diseases progression slowing Quality of life improvement
Prebiotics and Probiotics	Gut microbiota balance and SCFA production [161,176,177] Disorders prevention or management (obesity, diabetes mellitus, gastrointestinal disorders) [178,179,180,181,190,191,192,193,194] Pathogen inhibition (bacteriocins, competition for nutrients) [182] Intestinal barrier strengthening (modulation of mucin and pro-inflammatory cytokines production) [183,184] Stool features improvement [185,186,187,188,189] Behavior modulation (influence on GBA) [195,199,200] Gut–brain axis modulation (serotonin, GABA, dopamine, glutamate, signaling molecules) [324,325,326,327]	Stool quality improvement, beneficial shifting to fecal microbiota and metabolite profiles, blood lipids, and increased fecal IgA reduction [346] Gastrointestinal and immune health, as well as fecal quality metabolites improvement [343] Immune functions and antioxidant defense improvement [417,429,430,431,432,433,434] Overall physical condition, hair, and fecal quality improvement in cats [417] CKD markers improvement (BUN and creatinine concentrations reduction or preservation) and clinical symptoms improvement (appetite, activity, defecation frequency) [185] Stomatitis, gingivostomatitis, and oral infections reduction [193] Aggressiveness and anxiety decrease [191,199,200] Gastrointestinal pathologies (gastroenteritis and diarrhea) fast recovery [183,351,352,353,354] CADESI and PVAS score improvement [413,414] Leishmaniosis progression slowing [430] Gut microbiota improvement in dogs [344,349,350]	Cats with CKD at stage 2-3 [185] Dogs suffering from anxiety and aggression [191,199,200,372] Dogs suffering from exocrine pancreatic insufficiency [343] healthy household dogs [349], and cats [350] Dog puppies with gastroenteritis [351] and adults with diarrhea [183,352,353,354] Healthy adult dogs [346,347,429,431] Healthy cats [417,432] Dogs suffering from atopic dermatitis [413,414] Healthy adult castrated dogs [430] Leishmania infantum-infected dogs [434]	Gastrointestinal and extraintestinal health support and improvement Immunity status strengthening Chronic diseases progression slowing
Plant Extracts	Antioxidant and anti-inflammatory activity (TNF-α, IL-1β, and COX-2 reduction) [97,210,211,212,213,214,218] Immunomodulation [76,211] Antimicrobial and detoxifying activity [210,211] Cardioprotection and anticancer [76,210,211]	Lameness, willingness to move, play, and jump improvement [405,406] Intermittent lameness, local pain and stiff gait reduction in severity and resolution [225], Stress reduction [53] Metabolic profile improvement [104] lesions associated with granulomatous dermatitis (papules, nodules, plaques) and external otitis reduction [43] Behavioral issues (marking, anxiety, diffidence, irregular biorhythm, reactivity, activation, irritability, alertness, environmental exploration and attention requirement, fear, and hyperactivity) reduction [47,48] Antioxidant and immunological functions [354,435] enhancement CKS [44], epiphora [45], and leishmaniosis [52] progression slowing	Healthy dogs [104] Dogs with behavioral issues [47,48] Dogs suffering from chronic joint and spinal disease [225] Dogs suffering from mobility problems [405,406] dogs suffering from granulomatous dermatitis and external otitis [43] Adult and healthy dogs [354,435] Dogs suffering from CKS [44] and epiphora [45], infected by Leishmania infantum [52]	Quality of life improvement Immunity status strengthening
Dietary supplements	Anti-inflammatory and antioxidant defense (e.g., radical scavenging activity, synergism with other antioxidant molecules, influence of the activity of antioxidant enzymes, such as SOD) [224,228,231,234,290,292,301] Tissues and organs development [224,228,229] Coagulation process improvement [232,233] Cofactors or coenzymes in the metabolism of carbohydrates, proteins and lipids [225,226,227] Ensure the proper functioning of Antioxidant enzymes (SOD, alkaline phosphatase) [243,244] Ensure skeletal health and proper development of bones [251,252,253] Cancer prevention [262,263,264] Intracellular antioxidant, redox balance, maintenance, cofactors of antioxidant and detoxification enzymes [271,273,274,275] Toxic compounds and xenobiotics detoxification [269,272,286] Hepatoprotection [287] Cartilage protection [288,293]	Vision improvement [224] Skeletal and immune health promotion [229,230,241,242] Antioxidant defense improvement [433] Hair growth increase, superior hair quality, hair shedding decrease, and more hours of activity per day increase [415] Oxidative stress and related disease protection (hepatic, endocrine, GI) [259] Significant increases in erythrocyte total and reduced glutathione [277] SOD, catalase, glutathione peroxidase, total antioxidant capacity improvement [276] Xenobiotics detoxification (e.g., conjugation with GSH, enhancement of the excretion of toxic compounds from cells/body, direct neutralization of toxic compounds) [269,272] GSH erythrocytic levels and liver parameters (ALT, AST, ALP, GGT, BIL) improvement [287] scores for pain, weight-bearing and osteoarthritis severity reduction [304] Glycosaminoglycan synthesis increase and cartilage-degrading enzymes inhibition [288] Acetaminophen-induced toxicity reduction in cats [286]	Cats intoxicated with acetaminophen [286] Dogs suffering from liver disease [290] Dogs suffering from OA [304] Healthy dogs [438] Healthy senior dogs [415] Cats suffering from clinically evident and subclinical hypertrophic cardiomyopathy [276] Healthy senior cats [277]	Quality of life improvement Antioxidant defense improvement

## Data Availability

The original contributions presented in this study are included in the article. Further inquiries can be directed to the corresponding author.

## Abbreviations

The following abbreviations are used in this manuscript:

CR	caloric restriction
PUFAs	Polyunsaturated fatty acids
SC	short-chain
ALA	alpha-linolenic acid
LC	long-chain
EPA	Eicosapentaenoic acid
DHA	docosahexaenoic acid
NSAID	non-steroidal anti-inflammatory drug
AA	arachidonic acid
GFR	glomerular filtration rate
FOS	fructooligosaccharides
GOS	galactooligosaccharides
XOS	Xylooligosaccharides
SCFA	short-chain fatty acids
GLP-1	glucagon-like peptide-1
CKD	chronic kidney disease
BUN	blood urea nitrogen
GBA	gut–brain axis
NO	nitric oxide
IL	interleukin
COX	cyclooxygenase
TNF	tumor necrosis factor
SOD	superoxide dismutase
MBD	mineral and bone disease
GSH	reduced Glutathione
ROS	reactive oxygen species
GSSG	oxidized Glutathione
OS	oxidative stress
ALT	alanine transaminase
AST	alanine aminotransferase
ALP	alkaline phosphatase
GGT	gamma-glutamyl transferase
BIL	bilirubin
OA	osteoarthritis
DA	dopamine
5-HT	serotonin
GABA	gamma-aminobutyric acid
ENS	enteric nervous system
CNS	central nervous system
ASD	autism spectrum disorders
PD	Parkinson’s disease
AD	Alzheimer’s disease
BCFA	branched-chain fatty acids
TLR	toll-like receptor
IBD	inflammatory bowel disease
FMT	fecal microbiota transplantation
CBD	Canine Behavioral Checklist
C-BARQ	Canine Behavioral Assessment & Research Questionnaires
GHB	4-hydroxybutyric acid
BDNF	brain-derived neurotrophic factor
CDS	cognitive dysfunction syndrome
MMP	metalloproteinase
TMAO	trimethylamine-*N*-oxide
KO	Krill oil
NFHD	nonflea hypersensitivity dermatitis
CAD	canine atopic dermatitis
CAFR	cutaneous adverse food reaction
CPP	Chenpi powder
SIgA	secretory immunoglobulin A
WBC	white blood cell
MHC-II	major histocompatibility II complex
OTC	oxytetracycline
HCT	hematocrit
IMHA	immune-mediated anemia
KCS	keratoconjunctivitis sicca
SAG	S-acetyl-glutathione
IgG	Immunoglobulin G
